# Administrative Data Based Population Estimates for Scotland.

**DOI:** 10.23889/ijpds.v7i3.1816

**Published:** 2022-08-25

**Authors:** David Rowley, Caroline Ellis

**Affiliations:** 1 National Records of Scotland

In December 2021, we published statistical research on Administrative Data Based Population Estimates (ABPEs) for Scotland’s population in 2016, 2017 and 2018. This work was developed as part of a project to consider how administrative data could be used to support Scotland’s Census.

Following the governance process, administrative datasets were processed and de-identified, before being transferred to Scotland’s National Safe Haven for linking and analysis. The datasets used include data from health, the electoral register, vital events registrations, and education. The methodology used several linking variables so data could be linked, even without exact agreement between records. Records from across the data sources were resolved into individuals using these links. Business rules then indicated which individuals to include in Scotland’s Integrated Demographic Dataset (SIDD). The ABPEs were then produced from this and compared with the official mid-year population estimates (MYEs) to determine success.

On aggregate, the population estimates from the ABPEs are very similar to the MYEs, differing by less than 0.5 per cent in each year. When broken down further, larger differences occur with ABPEs having more males and fewer people aged over 65 when compared with the official statistics. A notable difference between the two is for males aged between 30 and 65 in deprived areas, with ABPEs up to 20% higher than the MYEs. These differences by deprivation are smaller for other age ranges and for females. The ABPEs tend to be higher than official estimates for urban areas, and lower for rural areas. Differences for each local authority area range from 5 per cent below to 4 per cent above official estimates.

It is therefore possible to produce Scottish population estimates purely from administrative sources that roughly agree with MYEs. Further investigation will help understand the differences for particular groups, and will be explored in future years by comparing ABPEs with the 2022 Census.

